# Structural Alternation in Heat Shock Proteins of Activated Macrophages

**DOI:** 10.3390/cells10123507

**Published:** 2021-12-11

**Authors:** Wenhao Zhang, Ying Wei, Huaijin Zhang, Jing Liu, Zhaoyun Zong, Zongyuan Liu, Songbiao Zhu, Wenxuan Hou, Yuling Chen, Haiteng Deng

**Affiliations:** MOE Key Laboratory of Bioinformatics, Center for Synthetic and Systematic Biology, School of Life Sciences, Tsinghua University, Beijing 100084, China; zwh17@mails.tsinghua.edu.cn (W.Z.); wy19@mails.tsinghua.edu.cn (Y.W.); zhang-hj16@mails.tsinghua.edu.cn (H.Z.); liuj17@mails.tsinghua.edu.cn (J.L.); zongzy@mail.tsinghua.edu.cn (Z.Z.); liuzy18@mails.tsinghua.edu.cn (Z.L.); zhusb14@mails.tsinghua.edu.cn (S.Z.); houwx19@mails.tsinghua.edu.cn (W.H.); chenyuling2016@mail.tsinghua.edu.cn (Y.C.)

**Keywords:** structural proteomics, RAW264.7, LPS, Lip-MS, HSP60

## Abstract

The inflammatory response of macrophages is an orderly and complex process under strict regulation accompanied by drastic changes in morphology and functions. It is predicted that proteins will undergo structural changes during these finely regulated processes. However, changes in structural proteome in macrophages during the inflammatory response remain poorly characterized. In the present study, we applied limited proteolysis coupled mass spectrometry (LiP-MS) to identify proteome-wide structural changes in lipopolysaccharide (LPS)-activated macrophages. We identified 386 structure-specific proteolytic fingerprints from 230 proteins. Using the Gene Ontology (GO) biological process enrichment, we discovered that proteins with altered structures were enriched into protein folding-related terms, in which HSP60 was ranked as the most changed protein. We verified the structural changes in HSP60 by using cellular thermal shift assay (CETSA) and native CETSA. Our results showed that the thermal stability of HSP60 was enhanced in activated macrophages and formed an HSP10-less complex. In conclusion, we demonstrate that in situ structural systems biology is an effective method to characterize proteomic structural changes and reveal that the structures of chaperone proteins vary significantly during macrophage activation.

## 1. Introduction

Macrophages play a major role in the host defense and inflammatory response. Macrophages can be activated by a wide range of cytokines and microbial ligands via the Toll-like receptor (TLR) signaling pathway [1,2,3]. When RAW264.7 macrophages are stimulated by lipopolysaccharide (LPS), the TLR4 signaling pathway is activated, which in turn activates the transcription factor nuclear factor κ B (NF- κB) and secretes different cytokines, such as tumor necrosis factor-α, IL1-β, and IL-6. Upregulation of cyclooxygenase 2 (COX2) and nitric oxide synthase (NOS) synthase and reactive oxygen species (ROS) production further exacerbate inflammation [4,5,6]. Although the process of macrophage activation has been well defined, the proteome-wide structural changes during macrophage activation have not been well characterized.

Limited proteolysis mass spectrometry (LiP-MS) is a powerful approach to characterize global structural changes [7,8]. Structural changes in proteins are attributed to (1) the binding of proteins to ligands, (2) protein–protein interactions, (3) protein post-translational modifications (PTM), or (4) protein aggregation [8]. An in-depth characterization of proteome-wide structural changes during macrophage activation will help us to have a deeper understanding of this process.

The 60-kDa heat shock protein (HSP60) is normally located in the mitochondria and plays an important role in the folding of imported mitochondrial proteins [9]. In mammals, HSP60 is usually present as a heptamer [10]. The HSP60 heptamer forms a complex with HSP10 in the presence of ATP to refold the misfolded proteins [11,12,13]. Models of the HSP60 polymorphic form have been proposed in previous studies, e.g., the football-type complex, the bullet-type complex, and the single-ring complex [10,14,15].

In the present study, we characterize the structural changes of the whole proteome in LPS-activated RAW264.7 by LiP-MS and provide a new resource for better understanding the inflammatory response.

## 2. Materials and Methods

### 2.1. Cell Culture and LPS Stimulation

The mouse macrophage cell line RAW264.7 was a generous gift from Xin Lin Laboratory, School of Medicine, Tsinghua University, Beijing, China. RAW264.7 cells were cultured in Dulbecco’s Modified Eagle Medium (DMEM) (Wisent, Nanjing, China) with 10% fetal bovine serum (FBS) (Wisent, Nanjing, China) and 1% penicillin/streptomycin (Wisent, Nanjing, China). Cells were plated in 10 cm dishes and cultured in an incubator containing 5% CO_2_ at 37 °C and, 95% relative humidity. After seeding for 24 h, cells were treated with *Escherichia coli* lipopolysaccharides (LPS, 100 ng/mL, L2880, Sigma, St. Louis, MO, USA) for 12 h or 24 h in the culture medium for macrophage activation.

### 2.2. Quantitative RT-PCR Assay

For quantitative RT-PCR, 2 × 10^5^ cells were used, and the total RNA from cell lysate was isolated using TRIzol extraction (TIANGEN BIOTECH, Beijing, China). Following RNA concentration measurement and assessment of its purity, equal amounts of total RNA (2 μg) from each sample were used for complementary DNA synthesis by using a reverse transcription system (Cwbio, Beijing, China) according to the manufacturer’s protocols. Quantitative RT-PCR was performed using SYBR Green reagent (Cwbio, Beijing, China). The relative standard curve method and 2(−ΔΔCt) method were used for quantitation and gene expression calculation, respectively. The sequences of primers used in this study are listed in Table S1.

### 2.3. Detection of Cellular Reactive Oxygen Species

The reactive oxygen species (ROS) levels in RAW264.7 after LPS stimulation for 2, 4, 6, 12, 18, and 24 h were detected using CellROX^®^ Deep Red Reagents (Thermo Fisher Scientific, Waltham, MA, USA) according to the manufacturer’s protocol. CellROX^®^ Deep Red Reagents were added at a final concentration of 5 μM and incubated at 37°C for 30 min. For each treatment, cells were analyzed on a BD FACSAria II Flow Cytometer (BD Biosciences, San Jose, CA, USA). Cellular ROS levels were detected in triplicate. Data were analyzed using Student’s *t*-test.

### 2.4. LiP-MS Analysis

LiP-MS experiments used untreated and LPS-treated RAW264.7 cells. Briefly, RAW264.7 cells were treated for 12 or 24 h with LPS treatment. Three 10-cm dishes (approximately 1 × 10^7^ cells per dish) were used for each treatment to serve as three technical repetitions. Cells were collected using a cell scraper, washed three times with PBS to remove the interference of serum protein in the culture medium, and then centrifuged at 400× *g* for 5 min to collect cells. After being centrifuged at 4 °C and 17,000× *g* for 5 min, the supernatant was quantified using the BCA protein assay kit (Thermo Fisher Scientific, Waltham, MA, USA) and diluted to 1 μg/μL. Finally, 400 μg of protein from each sample was divided into four PCR tubes for subsequent experiments. Two of them were used for LiP-digested samples and the other two tubes were used for trypsin-digested samples. All samples were placed in a 25 °C PCR instrument and preheated for 5 min. Then, 1 μg proteinase K (Sigma, St. Louis, MO, USA) per tube was added to the LiP-digested samples while 1 μg trypsin (Promega, Madison, WI, USA) per tube was added to the trypsin-digested samples. Sample tubes were placed in a 25 °C PCR instrument for 3 min and heated to 95 °C for 10 min to quench the digestion. Then, 100 μL of 10% sodium deoxycholate (Sigma, St. Louis, MO, USA) was added to each tube and heated at 95 °C for 10 min. Every two PCR tubes of the same digestion procedure were combined into one 1.5 mL tube. Next, 40 μL of 25 mM dithiothreitol was added and heated in a metal bath at 95 °C for 10 min. Then, 40 μL of 55 mM iodoacetamide was added at room temperature for 1 h in the dark to block the free sulfhydryl groups. Then, for all the samples, trypsin digestion was performed by adding 566 μL of 357 mM tetraethylammonium bromide (TEAB, Sigma, MO, USA), pH 8.0, and 2 μg trypsin (Promega, Madison, WI, USA) per tube, at 1200 rpm and 37 °C overnight. Afterwards, 20μL formic acid was used to acidify the samples, and the samples were centrifuged at 12,000× *g* for 10 min to remove the sodium deoxycholate precipitate. Sep-Pak C18 Vac cartridges (Waters, Milford, MA, USA) were used to desalt the samples. The eluted peptides were labeled overnight with tandem mass tags (TMT) 6-plex reagents (Thermo Fisher Scientific, Waltham, MA, USA), and the labeling reaction was quenched with 5% hydroxylamine. The TMT-labeled peptides were fractionated using a UPLC 3000 system (Thermo-Fisher Scientific, Waltham, MA, USA) equipped with an XBridge C18 RP column (Waters, Milford, MA, USA). Samples were separated into 48 fractions that were consolidated into 12 fractions and redissolved in 0.1% formic acid for LC-MS analysis.

### 2.5. LC-MS Analysis

The labeled peptides were analyzed by LC-MS/MS with nano-LC combined with an Orbitrap Fusion Lumos mass spectrometer. The digestion products were separated by a 120-min gradient elution at a flow rate of 0.30 μL/min using an UltiMate 3000 RSLCnano System (Thermo Fisher Scientific, Waltham, MA, USA), which directly interfaced with a Thermo Fusion Lumos mass spectrometer. The analytical column was a home-made fused silica capillary column (75 μm ID, 150 mm length; IDEX, Northbrook, IL, USA) packed with C18 resin (300 Å, 5 μm; Varian, Palo Alto, CA, USA). Mobile phase A consisted of 0.1% formic acid; mobile phase B consisted of 100% acetonitrile and 0.1% formic acid. The Fusion Lumos mass spectrometer operated in the data-dependent acquisition mode using Xcalibur 4.3 (Thermo Fisher Scientific, MA, USA) software, and there was a single full-scan mass spectrum in the orbitrap (350–1550 m/z, 120,000 resolution) followed by 3-sec cycles of data-dependent MS/MS scans at 34% normalized collision energy (HCD).

The MS/MS spectra from each LC-MS/MS run were searched against the selected database (Uniprot mouse reviewed proteome 7 March 2021) using in-house Proteome Discoverer 2.3 software (Thermo Fisher Scientific, Waltham, MA, USA). The search criteria were as follows: No-Enzyme (Unspecific) was required, carbamidomethylation (C) and TMT plex (K-terminal and *N*-terminal) were set as the fixed modifications, oxidation (M) and acetyl (protein *N*-terminal) were set as the variable modifications, the precursor ion mass tolerances were set at 10 ppm for all MS acquired in an orbitrap mass analyzer, and the fragment ion mass tolerance was set at 0.02 Da for all MS2 spectra acquired. Relative protein quantification was also performed using Proteome Discoverer software (version 2.3) according to the manufacturer’s instructions on the reporter ion intensities per peptide.

### 2.6. LiP-MS Data Analysis

The quantitative information of LiP-MS was analyzed by R language, and the R packages used were magrittr, tidyr, stringr, protti, tidyverse, ggthemes, ggrepel, dendextend, pheatmap, viridis, seriation, vioplot, GOplot, clusterProfiler, and dplyr. In brief, unique peptides were selected for quantitative calculation. All peptides with missing quantitative information were removed. All peptides from trypsin digestion or proteinase K digestion were normalized. The normalized reporter ion intensities from each replicate were used to calculate the Z-scores, which were converted into T-scores for ratio calculation. Considering that the protein abundance changes may affect the accuracy of the LiP peptide selection, we corrected the LiP peptide abundances with the protein fold changes. One-way analysis of variance (ANOVA) following Benjamini–Hochberg multiple testing (FDR = 5%) was used to evaluate the overall significance of the peptide and protein ratios; Tukey’s HSD was used to compare differences between the two groups [16]. Peptides or proteins analyzed with ANOVA and Tukey’s HSD adjusted *p*-value less than 0.05 were considered significantly changed (Figure S1a–d). Proteins with significantly changed LiP peptides were selected for GO enrichment analysis to draw chord mapping using the GOplot package.

### 2.7. Cellular Thermal Shift Assay (CETSA)

The CETSA experiment was performed according to the published method [17,18]. Briefly, cells were harvested, washed three times with PBS, resuspended in PBS containing 1× protease inhibitor, frozen in liquid nitrogen, centrifuged at 17,000× *g* to obtain cell lysates, stabilized at 25 ° C for 5 min, and then heated using a PCR instrument (Temperature gradient: 37, 40.4, 44, 46.9, 49.8, 52.9, 55.5, 58.6, 62, 66.3 °C, heating time: 3 min). The heated cell lysates were placed on ice for 10 min. The lysates were centrifugated at 100,000× *g* for 20 min using Max-XP (Beckman Optima, Brea, CA, USA), and the supernatant was subjected to Western blotting.

### 2.8. Western Blot Analysis

Protein samples obtained from the CETSA experiment above were loaded onto three 12% SDS-Page gels in 2× loading buffer and electro transferred onto polyvinylidenece difluoride (PVDF) membranes at 200 mA for 1 h. For the native CETSA analysis, 8–20% precast gel (Solarbio, Beijing, China) was used. The membranes were blocked in 5% milk in TBST buffer (20 mmol/L Tris-HCl, 150 mmol/L NaCl, and 0.1% Tween 20) for 1 h at room temperature. Each blocked membrane was incubated with primary antibodies against HSP60 (Cell Signaling Technology, Danvers, MA, USA, 1:2000, 12165S), HSP10(Abclonal, Woburn, MA, USA, 1:2000, A7437) and then HRP-conjugated secondary antibodies (1:2000 dilution, Cell Signaling Technology, Danvers, MA, USA). Samples were washed on the membranes with TBST three times, and the protein bands were visualized using an enhanced chemiluminescence reagent (Santa, Santa Cruz, CA, USA). To detect different antigens within the same blot, PVDF membranes were stripped with Restore Western Blot Stripping buffer and reprobed. Finally, the quantification of protein bands was performed by densitometry using Bio-Red Image lab 6.0.

## 3. Results

### 3.1. Modification of the LiP-MS Workflow for Characterization of the Structure-Ome

**Figure 1:**
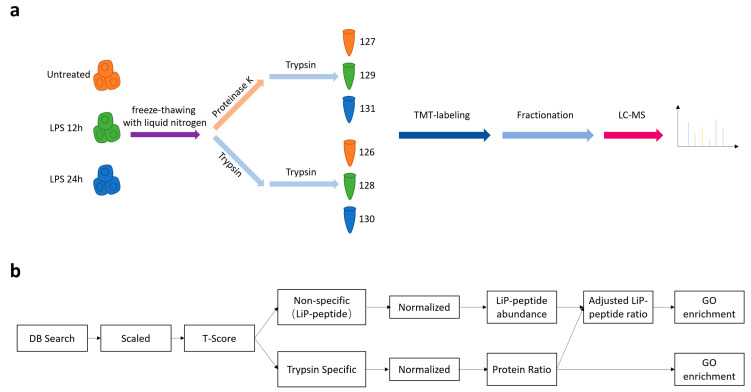
LiP-MS analysis for characterization of the structure-ome. (**a**) Workflow of LiP-MS analysis; (**b**) Workflow for LiP-MS data analysis.

We used LiP-MS to detect protein structural changes under LPS-induced inflammation. The original LiP-MS workflow was modified [8]. We obtained the cell lysates from untreated and LPS-treated cells by liquid nitrogen quick-freezing extraction. LiP experiments with proteinase K were performed to characterize the protein structural changes in an LPS-induced acute inflammation model. Peptides from trypsin digestion were used to characterize the changes in protein abundance. Then, we quantified the peptides generated by the LiP method with a TMT-6plex labeling reagent, which can relatively quantify six samples at the same time, allowing the detection of low abundance peptides [8,19] (Figure 1a). For the data analysis, we optimized the data processing workflow of LiP-MS. To reduce the error caused by changes in the protein content, we adjusted the peptide content of the LiP samples with the protein abundance change (Figure 1b).

### 3.2. Landscape of the Structure-Ome during Macrophage Activation

**Figure 2:**
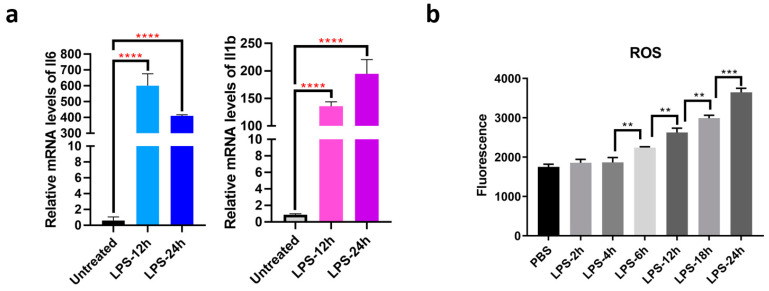
The production of ROS during macrophage activation. (**a**) Il-6 and Il-1β mRNA levels in RAW264.7 cells treated with LPS; (**b**) Detection of cellular ROS during macrophage activation. **: *p* < 0.01. ***: *p* < 0.001. ****: *p* < 0.0001.

**Figure 3:**
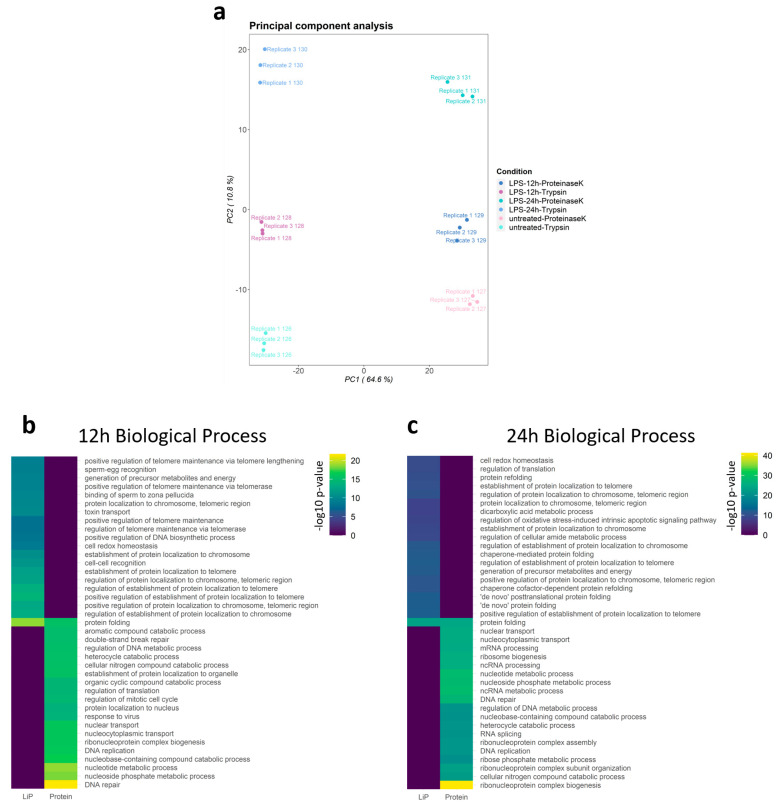
Landscape of the structure-ome during macrophage activation. (**a**) principal component analysis (PCA) of LiP-MS results; (**b**,**c**) Top 20 GO biological process enrichment comparison of protein abundance and structural changes after LPS stimulated for 12 h or 24 h.

We found that the mRNA levels of Il-6 and Il-1β in RAW264.7 cells significantly increased after LPS stimulation (Figure 2a). Through cellular ROS detection during macrophage activation, we selected 12 h and 24 h as the time points for our observation (Figure 2b). The LiP-MS results identified a total of 60,591 peptides belonging to 6365 proteins: 21,177 peptides were quantified in all channels, of which 6166 were LiP peptides and 15,011 were trypsin peptides. The principal component analysis (PCA) plot showed that the LiP-MS results were reproducible (Figure 3a). A total of 1925 proteins were differentially expressed, and 386 LiP peptides from 230 proteins were structurally changed in LPS-activated cells. The differentially expressed proteins and structurally-altered proteins were used for the subsequent GO-enrichment analysis. GO biological process enrichment analysis showed that differentially expressed proteins and structurally altered proteins were different, suggesting that LiP-MS provided structural information different from protein abundance changes (Figure 3b,c and Figures S2–S7).

### 3.3. GO Analysis of Structural Reads from Changed Proteins during Macrophage Activation

**Figure 4:**
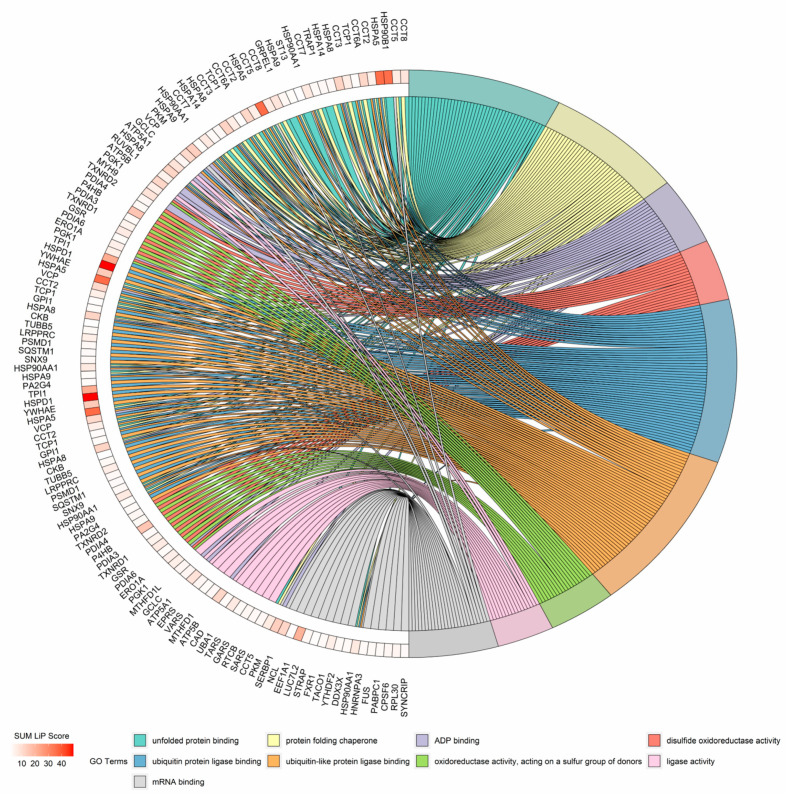
Gene ontology enrichment analysis of structural reads from significantly changed proteins during 24-h macrophage activation. The chord plot presents the linkages of genes and GO biological process terms.

We obtained the proteins with significant structural changes in LiP-MS experiments, and GO enrichment analysis was carried out for biological process enrichment analysis of these proteins. To show the extent of the structural changes detected, we summed the adjusted *p*-value of the LiP peptide ratios for each protein, named this the SUM LiP score, and presented our data in the form of a chord plot [20] (Figure 4). Chord plots show the top nine terms of the GO biological process. Protein folding was the most significant term of the biological process, ranked by adjusted *p*-value, containing *Hspd1*, *Hsp90b1*, *P4hb*, *Hspa8*, *Cct6a*, *Cct8*, *Hsp90aa1*, *Cct5*, and *Cct3* (Table 1). Protein folding is a process of assisting with the covalent and noncovalent assembly of single-chain polypeptides or multisubunit complexes into the correct tertiary structure. For the first time, we characterized the structural changes of protein folding in the inflammatory response. We chose the Hsp60 protein that had the highest SUM LiP score for further study of its structural changes.

### 3.4. Structural Changes in HSP60 during Macrophage Activation

**Figure 5:**
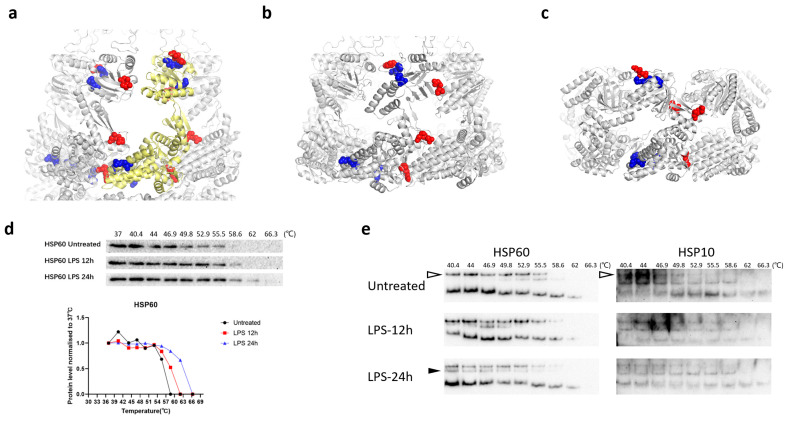
Structural changes in HSP60 during macrophage activation. (**a**) HSP60–HSP10 football-type complex. (PDB ID: 4PJ1); (**b**) HSP60–HSP10 bullet-type complex. (PDB ID: 6MRD); (**c**) HSP60 single-ring complex. (PDB ID: 7AZP) (Blue: Proteinase K cleavage site significantly decreased in LiP-MS analysis. Red: Proteinase K cleavage sites significantly increased in LiP-MS analysis. Pale yellow: HSP60 protein that has a different conformation to the other HSP60 proteins in this complex.); (**d**) CETSA analysis of HSP60; (**e**) native CETSA analysis of HSP60 and HSP10. (Hollow triangles indicate the complex formed by HSP60 and HSP10; the filled triangles indicate the complex formed by HSP60 without HSP10.).

HSP60 plays an important role in the maintenance of mitochondrial protein homeostasis during inflammation. HSP60 knockdown inhibits the inflammatory response during inflammation [21]. We color-marked the LiP peptides and LiP sites in three published human HSP60 structures by amino acid sequence alignment (Figure 5a–c and Figure S8) [9,13,14]. We verified whether HSP60 had significant structural changes by measuring its thermal stability after 24-h LPS treatment using CETSA assay (Figure 5d). The CETSA assay confirmed that the thermal stability of HSP60 was enhanced during macrophage activation. We found that significantly changed LiP sites were supportive of the transition of the HSP60 protein complex. This new type of complex prefers to dissociate HSP 10 and to undergo a transition from a bullet-type complex into a conformation similar to a single-ring complex. To confirm this hypothesis, we performed native CETSA analysis. HSP60 and HSP10 were observed to form a tight-binding complex in untreated cells, as observed on the native gel. After the 24-h LPS treatment, HSP60 dissociated from HSP10 to form a an HSP60-only complex (Figure 5e).

## 4. Discussion

In the present study, we characterized the structure-ome during macrophage activation and revealed changes in the complex state of the chaperone protein HSP60 during inflammation. The current study of protein structure is mainly focused on the structural analysis of the conformation in vitro, while the conformation of proteins in vivo is altered by their solvent environment, ligand binding or post-translational modification. There are only a few effective approaches that allow probing of in vivo proteome-wide protein structural changes. LiP-MS can characterize the structural changes of proteins in a complex cellular environment, which greatly deepens our understanding of the protein functions in biological processes. Compared to the thermal proteome profiling (TPP) that is a widely used method for identification of changes in ligand-induced protein conformation in both cell lysates or living cells, LiP-MS can provide site-specific structural changes with simple experimental procedures and low cost [18]. On the other hand, LiP-MS suffers low reproducibility and accuracy, and LiP-MS results need to be validated. Moreover, LiP-MS is mainly used to probe the structural changes of cytoplasmic proteins in cells. LiP-MS is becoming a useful tool in structural biology and can be applied to probe ligand- and posttranslational modification-induced protein conformation changes.

HSP60 is one of the major chaperone molecules that are ubiquitously expressed in all life forms [22]. HSP60 plays a crucial role in assisting with the folding, assembly, and transport of cellular proteins [23,24]. The mHsp60-mHsp10 complex assists in the refolding of denatured proteins [25,26]. Although various conformations including the football-type, bullet-type, and single-ring HSP60-HSP10 complex were observed with the crystallographic structural analysis or cryo-electron microscopy, there is still a lack of information on HSP60 structures during macrophage activation [10,14,15]. The LiP-MS results suggest that HSP60 undergoes conformational changes during macrophage activation. In the present study, we identified and validated that HSP60 exhibited a conformation change during macrophage activation. However, lack of access to advanced technology prohibited us from determining the biological functions of the HSP60 structural changes. We speculate that oxidative stress plays an important role in triggering HSP60 oxidation and structural changes. Advancement in technologies hold the key to deciphering in vivo structure–function relation.

In summary, we modified and applied LiP-MS to the identification of structure-ome changes in LPS-activated macrophages and found that heat shock proteins underwent conformational changes. Our results provide a valuable resource for understanding protein structure changes during macrophage activation.

## Figures and Tables

**Table 1:** Structural changes of protein folding-related genes after macrophage activation for 24 h.

Gene Names	Protein Description	24 h Sum LiP Score
Hspd1 Hsp60	60-kDa heat shock protein, mitochondrial	48.8701551
Hsp90b1	Heat shock protein 90-kDa beta member 1	35.57114236
P4hb	Endoplasmic reticulum resident protein 59	15.61354634
Hspa8 Hsc70 Hsc73	Heat shock cognate 71-kDa protein	11.97500326
Cct6a Cct6 Cctz Cctz1	T-complex protein 1 subunit zeta	11.27109261
Cct8 Cctq	T-complex protein 1 subunit theta	7.959656493
Hsp90aa1	Heat shock protein HSP 90-alpha	6.882919007
Cct5 Ccte	T-complex protein 1 subunit epsilon	6.202519596
Cct3 Cctg	T-complex protein 1 subunit gamma	5.911248248

## Data Availability

Mass spectrometry data have been uploaded to ProteomeXchange (PRIDE ID: PXD029672). The datasets for this study are available from the corresponding author upon reasonable request.

## Supplementary Materials

The following are available online at https://www.mdpi.com/article/10.3390/cells10123507/s1, Figure S1. The volcano plot of protein abundance and LiP peptide changes during the inflammatory response. (a) Protein abundance changes during 12-h LPS stimulation (Up:589; Down:523; ANOVA *p*.adjust ≤ 0.05; TukeyHSD *p*.adjust ≤ 0.05); (b) Protein abundance changes during 24-h LPS stimulation (Up:642; Down:470; ANOVA *p*.adjust ≤ 0.05; TukeyHSD *p*.adjust ≤ 0.05); (c) LiP peptide changes during 12-h LPS stimulation (Up:118; Down:61; ANOVA *p*.adjust ≤ 0.05; TukeyHSD *p*.adjust ≤ 0.05); (d) LiP peptide changes during 24-h LPS stimulation (Up:247; Down:109; ANOVA *p*.adjust ≤ 0.05; TukeyHSD *p*.adjust ≤ 0.05). Figure S2. LiP peptides enriched by GO Biological Process. Figure S3. LiP-peptide enriched by GO Molecular Function. Figure S4. LiP peptides enriched by GO Cellular Component. Figure S5. Proteins enriched by GO Biological Process. Figure S6. Proteins enriched by GO Molecular Function. Figure S7. Proteins enriched by GO Cellular Component. Figure S8. Sequence alignment of human HSP60 and mouse HSP60. Table S1. The sequences of primers used in this study. Table S2. The list of structural reads from significantly changed proteins in this study. Table S3. The list of LiP peptides significantly changed in 12-h LPS treatment. Table S4. The list of LiP peptides significantly changed in 24-h LPS treatment. Table S5. The list of proteins whose abundance significantly changed in 12-h or 24-h LPS treatment.
