# Supplementary material for: Structural Alternation in Heat Shock Proteins of Activated Macrophages
Source: Cells. 2021 Dec 11;10(12):3507. doi: 10.3390/cells10123507 (PMC8700196; doi:10.3390/cells10123507)
Supplement: Supplementary file 1 [file cells-10-03507-s001.zip › Figures S1-S8.pdf]

Supplementary Figure S1 The volcano plot of protein abundance and LiP-peptide changes during inflammatory response

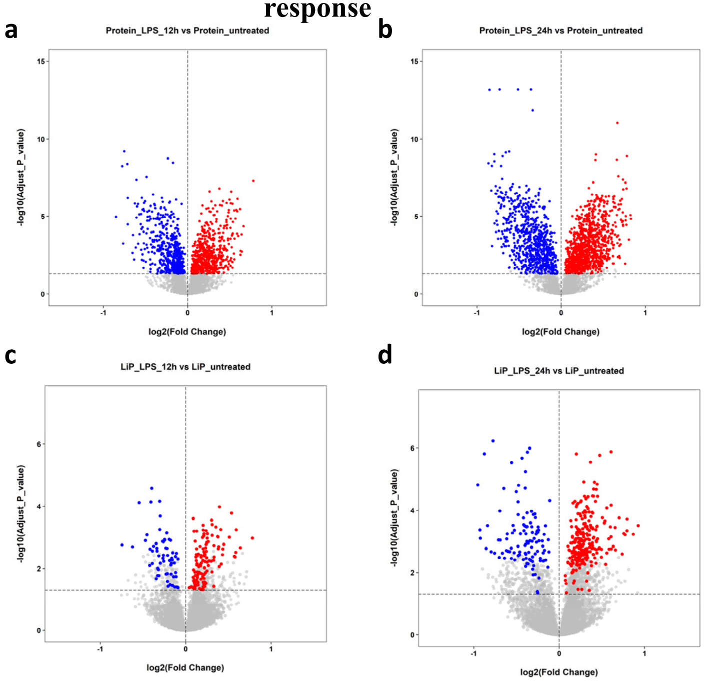

Supplementary Figure S2 LiP-peptide enriched by GO-Biological Process

LPS 12h

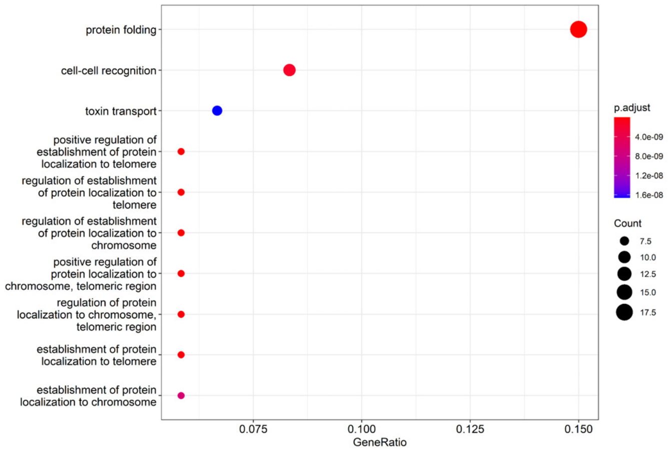

LPS 24h

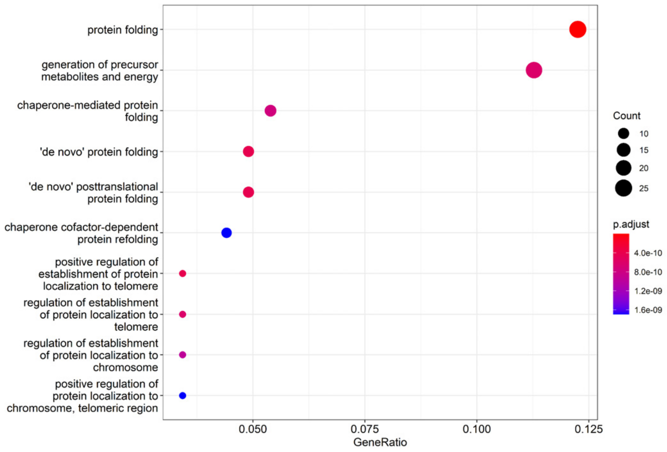

Supplementary Figure S3 LiP-peptide enriched by GO-Molecular Function

LPS 12h

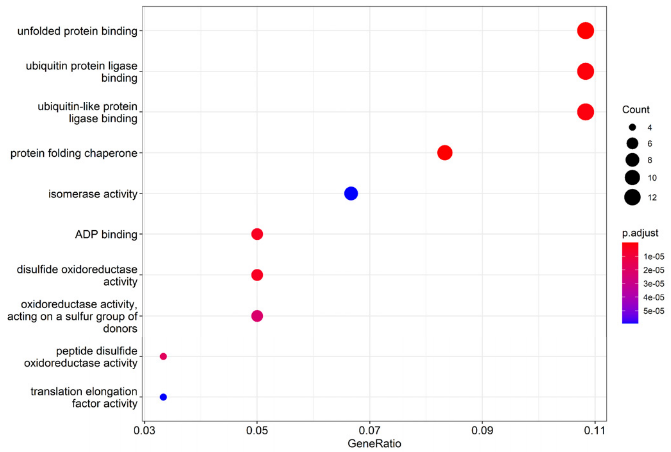

LPS 24h

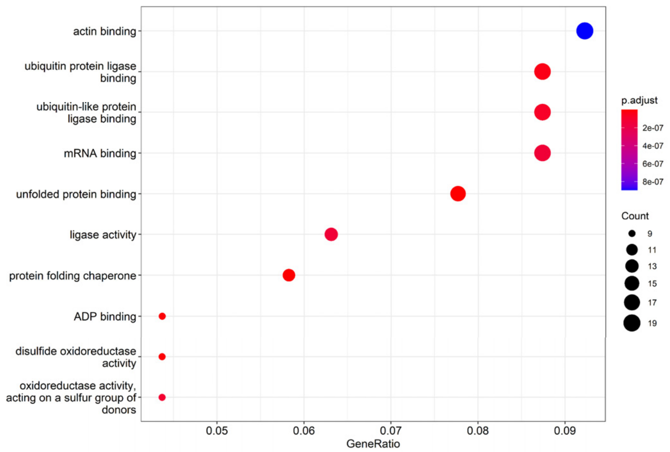

Supplementary Figure S4 LiP-peptide enriched by GO-Cellular Component

LPS 12h LPS 24h

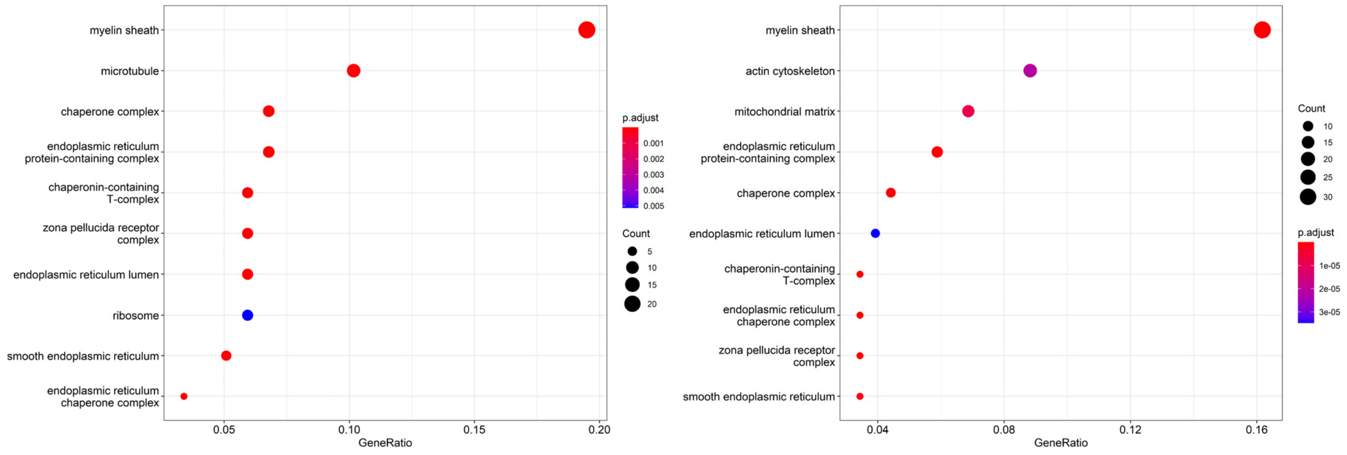

Supplementary Figure S5 Proteins enriched by GO-Biological Process

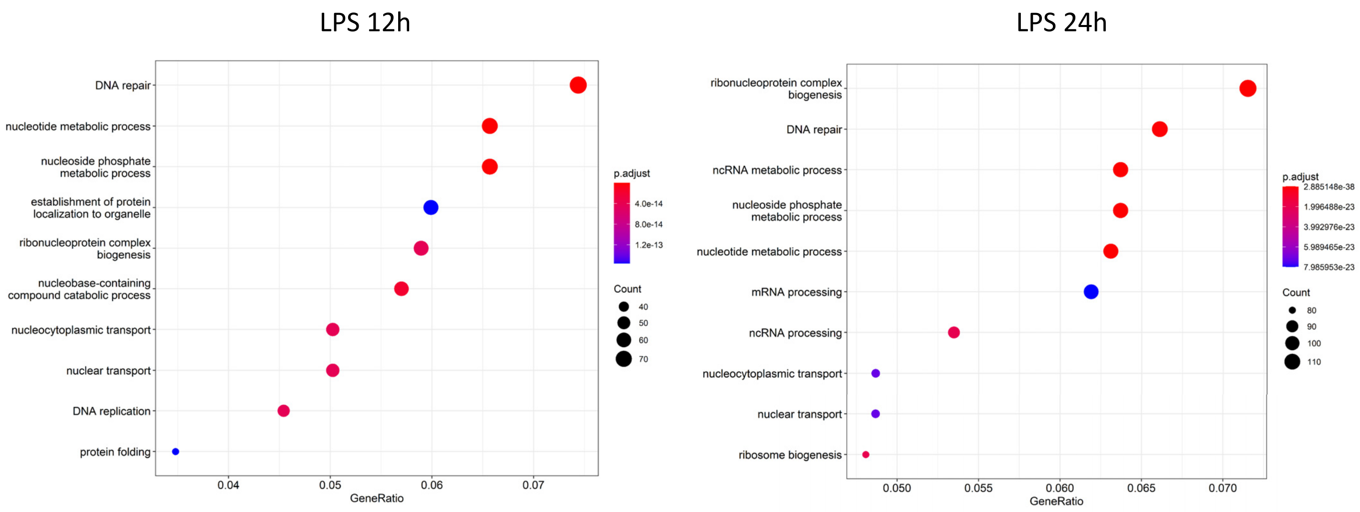

Supplementary Figure S6 Proteins enriched by GO-Molecular Function

LPS 12h

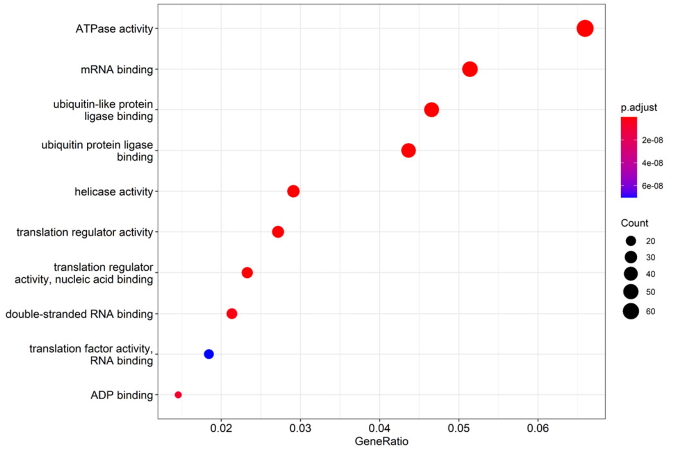

LPS 24h

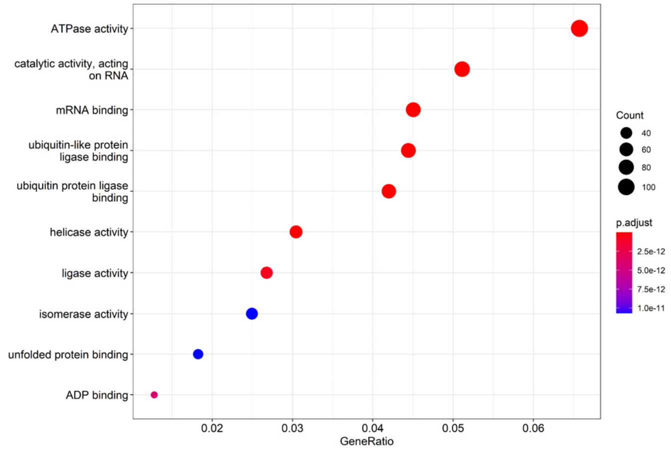

Supplementary Figure S7 Proteins enriched by GO-Cellular Component

LPS 12h

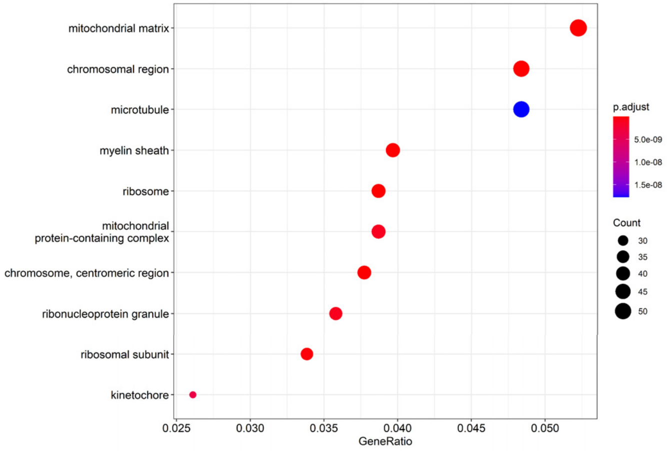

LPS 24h

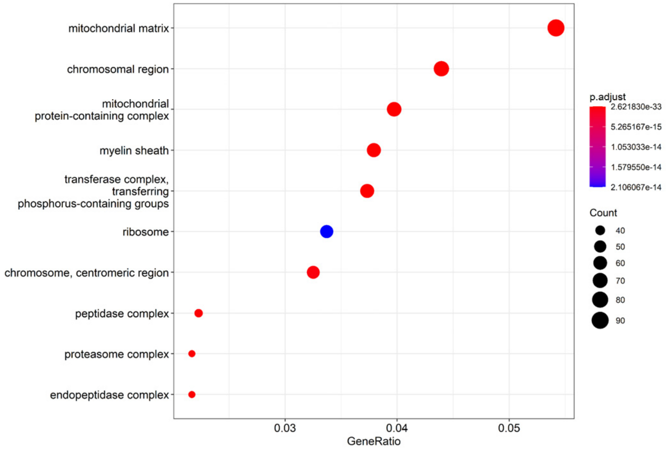

Supplementary Figure S8 Alinement of mouse HSP60 and human HSP60 protein sequences

```
P10809 CH60_HUMAN        1   MLRLPTVFRQMRPVSRVLAPHLTRAYAKDVKFGADARALMLQGVDLLADAVAVTMGPKGR
P63038 CH60_MOUSE        1   MLRLPTVLRQMRPVSRALAPHLTRAYAKDVKFGADARALMLQGVDLLADAVAVTMGPKGR
                             *******:*********.******************************************

P10809 CH60_HUMAN       61   TVIIEQSWGSPKVTKDGVTVAKSIDLKDKYKNIGAKLVQDVANNTNEEAGDGTTTATVLA
P63038 CH60_MOUSE       61   TVIIEQSWGSPKVTKDGVTVAKSIDLKDKYKNIGAKLVQDVANNTNEEAGDGTTTATVLA
                             ************************************************************

P10809 CH60_HUMAN      121   RSIAKEGFEKISKGANPVEIRRGVMLAVDAVIAELKKQSKPVTTPEEIAQVATISANGDK
P63038 CH60_MOUSE      121   RSIAKEGFEKISKGANPVEIRRGVMLAVDAVIAELKKQSKPVTTPEEIAQVATISANGDK
                             ************************************************************

P10809 CH60_HUMAN      181   EIGNIISDAMKKVGRKGVITVKDGKTLNDELEIIEGMKFDRGYISPYFINTSKGQKCEFQ
P63038 CH60_MOUSE      181   DIGNIISDAMKKVGRKGVITVKDGKTLNDELEIIEGMKFDRGYISPYFINTSKGQKCEFQ
                             :***********************************************************

P10809 CH60_HUMAN      241   DAYVLLSEKKISSIQSIVPALEIANAHRKPLVIIAEDVDGEALSTLVLNRLKVGLQVVAV
P63038 CH60_MOUSE      241   DAYVLLSEKKISSVQSIVPALEIANAHRKPLVIIAEDVDGEALSTLVLNRLKVGLQVVAV
                             *************:**********************************************

P10809 CH60_HUMAN      301   KAPGFGDNRKNQLKDMAIATGGAVFGEEGLTLNLEDVQPHDLGKVGEVIVTKDDAMLLKG
P63038 CH60_MOUSE      301   KAPGFGDNRKNQLKDMAIATGGAVFGEEGLNLNLEDVQAHDLGKVGEVIVTKDDAMLLKG
                             ******************************.******* *********************

P10809 CH60_HUMAN      361   KGDKAQIEKRIQEIIEQLDVTTSEYEKEKLNERLAKLSDGVAVLKVGGTSDVEVNEKKDR
P63038 CH60_MOUSE      361   KGDKAHIEKRIQEITEQLDITTSEYEKEKLNERLAKLSDGVAVLKVGGTSDVEVNEKKDR
                             *****:******** ****:****************************************

P10809 CH60_HUMAN      421   VTDALNATRAAVEEGIVLGGGCALLRCIPALDSLTPANEDQKIGIEIIKRTLKIPAMTIA
P63038 CH60_MOUSE      421   VTDALNATRAAVEEGIVLGGGCALLRCIPALDSLKPANEDQKIGIEIIKRALKIPAMTIA
                             **********************************.***************:*********

P10809 CH60_HUMAN      481   KNAGVEGSLIVEKIMQSSSEVGYDAMAGDFVNMVEKGIIDPTKVVRTALLDAAGVASLLT
P63038 CH60_MOUSE      481   KNAGVEGSLIVEKILQSSSEVGYDAMLGDFVNMVEKGIIDPTKVVRTALLDAAGVASLLT
                             **************:*********** *********************************

P10809 CH60_HUMAN      541   TAEVVVTEIPKEEKDPGMGAMGGMGGGMGGGMF
P63038 CH60_MOUSE      541   TAEAVVTEIPKEEKDPGMGAMGGMGGGMGGGMF
                             ***.*****************************
```
